# Financial hardship experience in middle- and older-aged patients with advanced lung cancer

**DOI:** 10.1007/s00520-024-08571-7

**Published:** 2024-05-22

**Authors:** Naomi Takemura, Shumin Jia, Chia-Chin Lin

**Affiliations:** 1 School of Nursing, Li Ka Shing Faculty of Medicine, Academic Building, The University of Hong Kong, 5/F3 Sassoon Road, Pokfulam, Hong Kong; 2 Alice Ho Miu Ling Nethersole Charity Foundation Professor in Nursing, Pokfulam, Hong Kong

**Keywords:** Correlates, Financial hardship, Health outcomes, Lung cancer

## Abstract

**Purpose:**

Advancements in medical treatments have resulted in increased medical costs for cancer patients. More than half of the patients with advanced lung cancer reported unmet financial needs. The purpose of this study is to examine the differences in the prevalence and correlates of financial hardship between middle- and older-aged patients with advanced lung cancer, and its impact on multiple health-related outcomes.

**Methods:**

This study presents a cross-sectional analysis involving 226 patients with advanced lung cancer, who were enrolled in a randomized controlled trial conducted between 2018 and 2020. Data collection was performed through self-reported questionnaires and electronic medical records. Multivariable logistic and linear regression models were adopted for analysis.

**Results:**

58.0% reported experiencing financial hardships. Middle-aged participants who were single and had a lower education level were more likely to experience financial difficulties. However, males and higher performance status were associated with a lower likelihood of experiencing financial difficulties among older-aged participants. Financial hardship was significantly associated with anxiety (*p* < 0.001), depression (*p* < 0.001), sleep disturbances (*p* < 0.001), quality of life, global health status (*p* = 0.002), functional scale score (*p* < 0.001), symptom scale score (*p* < 0.001), and lung cancer-specific scale score (*p* < 0.001).

**Conclusions:**

More than half of the patients with advanced lung cancer experienced financial hardships caused by cancer or its treatment, with a higher prevalence reported in middle-aged patients. Different sociodemographic and clinical variables correlated with financial hardship in middle- and older-aged participants, respectively. More attention should be paid to middle-aged patients with advanced lung cancer, particularly during routine assessments.

## Background

Lung cancer is one of the most common and deadliest types of cancer worldwide [1]. Specifically, lung cancer has been consistently ranked as the leading cancer diagnosis and cause of cancer death in Hong Kong since 2019 [2]. Unfortunately, approximately 70% of lung cancer patients are diagnosed at an advanced stage [2]. With advances in cancer treatment, lung cancer survival has continuously increased [3, 4], along with expenditures on cancer treatment. In addition to the high level of physical and psychological symptom burden, lung cancer, particularly advanced stage cancer, has a substantial financial impact on patients and their families, including both direct and indirect costs, such as reduced employment (reduced hours and work productivity) during cancer treatment [5, 6]. More than half of the patients with advanced lung cancer reported unmet financial needs [7]. Financial hardship is an established side effect of cancer treatment, and its influence on clinical outcomes and experiences of care for patients with cancer has been an ongoing area of interest in healthcare research [8].

Based on the financial hardship typology, there are three domains of financial hardship. Material conditions arise from increased out-of-pocket expenses as well as the potentially lower income, because of the inability to work during or after cancer treatment [9]. Psychological responses arise from feelings of distress owing to the costs of cancer treatment and care. Coping behaviors, such as delaying, reducing, or skipping cancer treatment, arise because of the inability to afford such medical costs [10]. All these domains have a negative impact on health outcomes, leading to a deteriorated quality of life and an increased risk of death [11].

A recent study revealed that an increase in financial toxicity was moderately associated with a decline in quality of life among patients with advanced lung cancer in Western China [12], whereas increasing financial toxicity was significantly associated with shorter progression-free survival, but not overall survival, in patients with locally advanced lung cancer [13]. Given the association between financial hardship and poor health outcomes, it is imperative to better comprehend the prevalence, correlates, and impact of financial hardship experienced by patients and their families, to provide effective cancer care and promote their financial well-being. Younger age has been reported as a common risk factor for high financial toxicity in patients with advanced lung cancer in Western and non-Western countries [12, 14]. To date, no studies have examined financial hardships in middle-and older-aged patients with advanced lung cancer. Anxiety and psychological distress are common psychoneurological symptoms that co-occur in patients with lung cancer [15]. Hence, this study aimed to examine the differences in the prevalence and correlates of financial hardship between middle- and older-aged patients with advanced lung cancer, and its impact on multiple psychoneurological symptoms and overall survival.

## Methods

### Study design and participants

This was a secondary data analysis of the baseline data from a large-scale randomized controlled trial investigating the effectiveness of physical activity on sleep quality and other associated bio-physiopsychological outcomes in patients with advanced lung cancer (ClinicalTrials.gov Identifier: NCT04119778) [16]. The eligibility criteria were based on the primary study [16]. Participants were recruited if they were diagnosed with stage IIIB or IV non-small cell lung cancer, without other cancer diagnoses within the previous year, with an Eastern Cooperative Oncology Group Performance Status of 0–2, aged at least 18 years, and reported not engaging in regular exercise. Patients with neurological or psychiatric disorders were excluded.

### Procedures

Participants were recruited from three major hospitals in Hong Kong between December 2018 and September 2021, and recruitment procedure has been paused for six months due to the COVID-19 pandemic. Research personnel approached the potential participants at the out-patient clinics of the three hospitals and interested patients provided written informed consent. Ethical approval was obtained from the Institutional Review Board of the University of Hong Kong/Hospital Authority Hong Kong West Cluster (UW 18–154), Hong Kong East Cluster (HKECREC-2019–014), and Kowloon Central Cluster/Kowloon East Cluster (KC/KE-19–0039/ER-3). The study was conducted following the Declaration of Helsinki.

### Outcome measures

#### Financial difficulty

Financial difficulty was measured using question 28 from the European Organization for Research and Treatment of Cancer Quality of Life Core Questionnaire (EORTC QLQ-C30) [17], “During the last week, has your physical condition or medical treatment caused you financial difficulties?” The item was rated on a 4-point Likert scale, with 1 denoting “not at all” and 4 denoting “very much.”

#### Sleep disturbance

Sleep disturbance was measured using the Pittsburgh Sleep Quality Index (PSQI), which consists of 19 items across seven components (subjective sleep quality, sleep latency, sleep duration, habitual sleep efficiency, sleep disturbances, use of sleeping medication, and daytime dysfunction) rated on a scale ranging from 0 to 3. The total score ranged from 0 to 21, with higher scores indicating poorer sleep. The PSQI has been validated in the Chinese population [18].

#### Anxiety and depression

Anxiety and depression were measured using the Hospital Anxiety and Depression Scale (HADS), which consists of two subscales: anxiety and depression. Each subscale comprises seven items rated on a 4-point scale. A cutoff score of ≥ 8 on either subscale indicated anxiety or depression [19]. HADS has been validated in a Chinese cancer population [20].

#### Quality of life

Health-related quality of life is an important outcome for patients with lung cancer and has a substantial impact on their prognosis. The Chinese version of the EORTC QLQ-C30 (30 items) and the corresponding lung cancer-specific module (QLQ-LC13:13 items) were used to measure quality of life. The EORTC QLQ-C30 covers general aspects of health-related quality of life [17], and three quality of life scales from the QLQ-C30 were included in the analysis: global health status, functional status, and symptom scale. The QLQ-LC13 module comprises items measuring lung cancer-associated symptoms and the side effects of conventional chemotherapy and radiotherapy [21], and one symptom scale was included in the analysis. The EORTC is a valid and reliable measure of quality of life, used in Chinese patients with lung cancer [22].

#### One-year survival rate

The duration of survival, including information on patients who had passed away, was retrieved from electronic medical records in the hospitals.

#### Demographic and clinical factors

Sociodemographic variables, cancer-related information, and lifestyle factors were assessed using a self-designed questionnaire. The sociodemographic variables included age, sex, education, and marital status. Cancer-related information included current treatment modalities (targeted or non-targeted therapy) and time since diagnosis (months). Lifestyle factors included smoking (smoker or non-active smoker) and drinking (drinker or non-active drinker) behaviors. Nurses assessed the Karnofsky Performance Status (KPS) score, which measures the level of patient activity and independence [23].

### Statistical analysis

Descriptive statistics were used to summarize the sociodemographic, clinical, and treatment characteristics of the participants and the prevalence of financial hardships. Continuous variables were summarized as mean ± standard deviation (SD), and categorical variables were summarized as counts and percentages. Multivariate logistic regression analysis was used to assess the association between sociodemographic and clinical variables (sex, education, marital status, KPS score, time since diagnosis, and treatment modality), and the presence of financial hardship among middle-and older-aged patients, respectively. Multivariate linear regression analysis was used to assess the association between financial hardship and numerous psychosocial outcomes (anxiety, depression, sleep disturbance, quality of life, and survival). For all models, the data were checked for the absence of multicollinearity (assessed using the variance inflation factor [VIF]), homoscedasticity, and the normal distribution of residuals. There were no missing data to be handled. All analyses were conducted with the SPSS, v.25.0, IBM Corporation, and were two sided. Statistical significance was defined as a *p*-value < 0.05.

## Results

### Characteristics of study participants

Table 1 presents the characteristics of the study participants. Among the 226 participants, 148 were aged 65 years or younger and 78 were aged above 65 years. More than half (54.0%) was female. The majority were married or cohabiting (77.4%), had secondary education or above (85.9%), were non-active smokers (94.7%), and non-active drinkers (92.5%). The participants were diagnosed with cancer for a mean duration of 25.32 months. The vast majority (98.2%) of the participants were undergoing cancer treatment. There were significant differences in education level (*p* = 0.017), financial difficulty (*p* = 0.048), and employment status (*p* < 0.001) between the middle-aged (those aged 65 or below) and older-aged (those aged above 65).

**Table 1:** Characteristics of study participants

	Participants, no (%)			
Characteristic	All (*N* = 226)	Middle-aged (≤ 65 years old) (*N* = 148)	Older aged (> 65 years old) (*N* = 78)	*p* value
Sex				0.577
Male	104 (46.0)	66 (44.6)	38 (48.7)	
Female	122 (54.0)	82 (55.4)	40 (51.3)	
Marital status				0.738
Married or cohabiting	175 (77.4)	116 (78.4)	59 (75.6)	
Single	51 (22.6)	32 (21.6)	19 (24.4)	
Education				0.017*
Illiterate to primary	32 (14.2)	14 (9.5)	18 (23.1)	
Secondary	143 (63.3)	97 (65.5)	46 (59.0)	
Tertiary or above	51 (22.6)	37 (25.0)	14 (17.9)	
KPS, mean (SD)	89.73 (7.18)	89.93 (7.3)	89.36 (6.9)	0.569
Months since diagnosis, mean (SD)	25.32 (31.35)	24.64 (29.9)	26.62 (34.0)	0.652
Treatment modalities				0.220
Targeted therapy	133 (58.8)	89 (60.1)	44 (56.4)	
Non-targeted therapy	89 (39.4)	58 (39.2)	31 (39.7)	
No treatment	4 (1.8)	1 (0.7)	3 (3.8)	
Employment				< 0.001*
Employed	51 (22.6)	45 (30.4)	6 (7.7)	
Unemployed	175 (77.4)	103 (69.6)	72 (92.3)	
Financial hardship				0.048*
No	95 (42.0)	55 (37.2)	40 (51.3)	
At least a little	131 (58.0)	93 (62.8)	38 (48.7)	

Abbreviation. *KPS*, Karnofsky Performance Status; *SD*, standard deviation

### Prevalence of financial difficulties

Among the 226 participants, 58.0% (*n* = 131) reported experiencing minimal financial difficulties (Table 1). The prevalence of financial difficulty was higher among middle-aged individuals (62.8%) than among older adults (48.7%). By contrast, the prevalence of unemployment was higher among older adults (92.3%) than among middle-aged adults (69.6%).

### Correlates of financial difficulties in middle-and older-age patients

Table 2 presents the results of the multivariate linear regression analysis used to identify the sociodemographic and clinical variables associated with financial difficulties among middle- and older-aged participants. Among middle-aged participants, being single (OR = 4.056, 95% CI = 1.394, 11.804, *p* = 0.010) and illiterate, or having primary school (OR = 5.750, 95% CI = 1.236, 26.760, *p* = 0.026) and secondary school (OR = 2.313, 95% CI = 1.013, 5.278, *p* = 0.046) education level compared to tertiary or above were significantly associated with higher risk of experiencing financial difficulties. Among older adults, a higher KPS score (OR = 0.871, 95% CI = 0.784–0.969, *p* = 0.011) and male sex (OR = 0.311, 95% CI = 0.105–0.919, *p* = 0.035) were significantly associated with a lower risk of financial difficulties.

**Table 2:** Logistic regression models for correlates of financial difficulties in middle-and older-aged participants

	Middle-aged participants (≤ 65 years old)		Older-aged participants (> 65 years old)	
Variables	OR (95% CI)	*p*	OR (95% CI)	*p*
Gender				
Male (vs. female)	1.047 (0.481, 2.281)	0.908	0.311 (0.105, 0.919)	0.035*
Education				
Illiterate to primary school (vs. tertiary or above)	5.750 (1.236, 26.760)	0.026*	0.970 (0.188, 5.003)	0.971
Secondary school (vs. tertiary or above)	2.313 (1.013, 5.278)	0.046*	1.707 (0.397, 7.330)	0.472
Marital status				
Single (vs. married or cohabiting)	4.056 (1.394, 11.804)	0.010*	2.092 (0.618, 7.088)	0.236
KPS	0.984 (0.937, 1.034)	0.519	0.871 (0.784, 0.969)	0.011*
Time since diagnosis	1.001 (0.989, 1.014)	0.827	0.988 (0.971, 1.004)	0.135
Treatment modality				
Targeted therapy (vs. non-targeted therapy)	0.534 (0.219, 1.304)	0.169	0.891 (0.271, 2.923)	0.849

Abbreviation. *CI*, confidence interval; *KPS*, Karnofsky Performance Status

* *p* < 0.05

### Relationship between financial difficulties and psychosocial factors

Table 3 presents the results of the multivariate linear regression analyses used to examine the relationship between financial difficulties and psychosocial factors. After adjusting sociodemographic and clinical variables (sex, education, marital status, smoking, drinking, KPS, time since diagnosis, and treatment modality), the presence of financial difficulty was significantly associated with anxiety (*β* = 3.200, 95% CI = 2.047, 4.353, *p* < 0.001), depression (*β* = 2.018, 95% CI = 0.638, 3.398, *p* < 0.001), sleep disturbances (*β* = 1.789, 95% CI = 0.768, 2.810, *p* < 0.001), quality of life global health status (*β* =  − 10.376, 95% CI =  − 16.879, − 3.872, *p* = 0.002), functional scale (*β* =  − 10.834, 95% CI =  − 14.961, − 6.706, *p* < 0.001), symptom scale (*β* = 12.307, 95% CI = 8.499, 16.114, *p* < 0.001), and lung cancer-specific scale (*β* = 11.035, 95% CI = 6.261, 15.810, *p* < 0.001). The VIF for all factors was less than 5, indicating the absence of multicollinearity.

**Table 3:** Linear regression model for factors associated with financial difficulty among middle-aged participants (*n* = 148)

	Multivariable regression^a^	
Variables	Beta (95% CI)	*p*
HADS-Anxiety	3.200 (2.047, 4.353)	< 0.001*
HADS-Depression	2.018 (0.638, 3.398)	< 0.001*
PSQI	1.789 (0.768, 2.810)	< 0.001*
QOL-Global health status	− 10.376 (− 16.879, − 3.872)	0.002*
QOL-Functional scale	− 10.834 (− 14.961, − 6.706)	< 0.001*
QOL-Symptom scale	12.307 (8.499, 16.114)	< 0.001*
QOL-Lung cancer module	11.035 (6.261, 15.810)	< 0.001*
Adherence	0.074 (− 0.052, 0.199)	0.249
One-year survival	0.069 (− 0.131, 0.268)	0.497

^a^Covariates: Gender, body mass index, education, marital status, KPS, time since diagnosis, and treatment modality

Abbreviation. *CI*, confidence interval; *HADS*, Hospital Anxiety and Depression Scale; *PSQI*, Pittsburgh Sleep Quality Index; *QOL*, quality of life

## Discussion

This is the first study to identify the prevalence and correlates associated with financial hardship among middle- and older-aged patients with advanced lung cancer, as well as their impact on multiple health-related outcomes and overall survival. In our sample, more than half (58%) of the participants experienced financial difficulties, with a higher prevalence among middle-aged participants. Different sociodemographic and clinical variables were correlated with financial difficulties in the middle-and older-aged participants, respectively. Notably, financial difficulties are significantly associated with psychological distress, sleep disturbance, and quality of life.

Financial difficulties are prevalent among patients with cancer, especially owing to the development of more effective therapies that have gradually been applied in clinical practice [24]. Our findings revealed that the prevalence of financial difficulty related to cancer and its treatment was 58% in patients with advanced lung cancer in Hong Kong, which is similar to that found in patients with lung cancer in the United States being 51.9–61.0% [8, 25, 26] but lower than that of their Chinese counterparts (83.7%) [27]. Hong Kong’s public healthcare system is available to citizens of all ages regardless of their financial status. Oncology centers in the public healthcare system provide comprehensive anticancer therapies, including chemotherapy, radiotherapy, targeted therapies, and brachytherapy, at an affordable cost. However, cancer patients must pay out-of-pocket for the latest treatment options, such as newly developed targeted therapies and immunotherapies, which are more expensive and not subsidized by the public healthcare system [28]. The self-financed cancer treatments were accessible in both public and private hospital sectors, as well as in community pharmacies [29]. Additionally, cancer patients must pay for regular imaging tests, such as PET scans, CT scans, and MRIs, outside public healthcare settings. These imaging tests were readily available in private clinics or hospitals. As of 2021, merely 49.7% of the population in Hong Kong possessed medical insurance coverage [30]. Hence, the financial burden on patients with cancer in Hong Kong is considerable. Notably, the prevalence of financial difficulty was higher among middle-aged adults than among older adults. Previous studies on lung cancer reported that younger age was a risk factor and associated with a greater level of financial burden in both Western and non-Western countries [12, 14, 31], indicating that more attention should be paid to the younger generation of cancer survivors regarding their financial status. In Western countries, this could be explained by the fact that patients younger than 65 years were not benefitted from “universal” health coverage for all prescription of medications [14], whereas, in Hong Kong, this situation could be attributed to the Chinese social phenomenon of middle-aged adults taking care of the older (parents) and the younger (children) generations. Being diagnosed with cancer, particularly late-stage cancer, could have resulted in unemployment or being employed but was unpaid [32], adding additional psychological and financial burden to middle-aged patients.

Different sociodemographic and clinical variables were significantly correlated with financial difficulties in the middle-and older-aged participants, respectively. In line with a previous study on patients with advanced cancer, being single was associated with a higher likelihood of financial difficulties [33], as they might not have partners to support their daily living or medical expenditures, and middle-aged patients were yet to receive pensions from the government. Additionally, patients with lower levels of education are likely to experience financial difficulties, which could be attributed to the fact that family income is inversely related to the educational level in patients with advanced cancer [34]. In contrast to middle-aged patients, male sex and better performance status were associated with a lower likelihood of financial difficulties in older-aged participants. The KPS was found to have a moderate negative correlation with multiple symptoms in older patients with cancer, which could increase the symptoms and financial burden in this group of patients [35].

Expanding on studies in the United States and Western China reporting a significant correlation between financial toxicity and health-related quality of life among patients with lung cancer [12, 36], our study revealed that the presence of financial difficulties was significantly associated with a deteriorated quality of life and higher levels of anxiety, depression, and sleep disturbance. This finding reflects the negative health impact of experiencing financial toxicity, especially among this vulnerable population, who is susceptible to a more severe level of symptom burden than other cancer patients. This implied the presence of a vicious cycle in which patients with a higher symptom burden were less likely to be able to return to work [37], and the problem of financial burden was less likely to be solved. Additionally, Lathan et al. [8] found that financial strain was continuously associated with negative changes in the quality of life and symptom burden in patients with lung and colorectal cancer, 12 months after diagnosis. The average time of diagnosis in our participants was approximately 2 years, suggesting that the existence of financial difficulties is a long-term problem and would not be reduced with the increase in the time of diagnosis. The negative health impact of financial difficulties and their continuous existence throughout the cancer trajectory highlight the need to identify patients with financial difficulties at an earlier stage, and implement timely interventions to address this problem.

### Limitation

The strength of this study is that it included a specific and understudied population with the most common cancer type worldwide. It examined the impact of financial hardship on multiple health outcomes in patients with advanced lung cancer. However, this study has several limitations. First, it used a single question from the EORTC QLQ-C30, a validated tool for health-related quality of life, to assess financial hardship. Although previous studies have used it to evaluate financial hardship, future studies could adopt validated tools such as the Comprehensive Score for Financial Toxicity to assess financial distress. Second, this study is limited by its cross-sectional design. Future studies could employ a longitudinal design to examine the trajectory and dynamic characteristics of financial hardship experienced by patients throughout lung cancer survivorship, as the financial consequences of cancer are long-lasting. Third, participants’ profession was not measured, which has the potential to affect the probability of lung cancer diagnosis.

### Implication for future research and practice

Our study has several implications for future research and practice. The findings highlight the need to develop and evaluate interventions to address the financial difficulties faced by patients with advanced lung cancer. Interventions such as financial counseling programs that provide patients with information on financial assistance programs, insurance coverage, or financial incentives for adhering to medical treatment could aid in reducing out-of-pocket costs. However, further research is required to evaluate the effectiveness of these interventions. Additionally, this study highlights the need for a multidisciplinary approach to assess and address the financial difficulties of patients with advanced lung cancer. This may include collaborations among various healthcare professionals, patient advocacy groups, insurers, and policymakers, to assess, develop, and implement comprehensive interventions that address the direct and indirect costs of cancer care. By implementing a patient-centered approach, healthcare providers can pay more attention to middle-aged patients who are single and have lower education levels, as well as to older-aged patients with lower performance status, when performing initial assessment. Additionally, healthcare providers can improve the quality of life and health outcomes of patients and their caregivers by alleviating their financial burdens. For example, expanding access to health insurance coverage, minimizing out-of-pocket costs for cancer treatment, supporting paid sick leave, and arranging flexible working arrangements could help manage the direct and indirect costs of cancer care.

## Conclusion

In summary, our findings highlight that more than half of the patients with advanced lung cancer experience financial difficulties caused by their physical condition or medical treatment. The prevalence was higher in middle-aged than older-aged participants. Lower educational levels and being single were found to correlate with financial difficulties in middle-aged participants, whereas male sex and higher performance status were associated with a lower likelihood of financial difficulties in older-aged participants. Additionally, financial difficulties negatively impact multiple health-related outcomes in this population, namely psychological distress, sleep disturbances, and quality of life. Future research is warranted to routinely assess and comprehend financial difficulties in this population based on the abovementioned correlates, and thereby develop measures and interventions to address this issue.

## Data Availability

The datasets used and/or analyzed during the current study are available from the corresponding author on reasonable request.
